# Efficiency of different control measures for preventing carbapenemase-producing enterobacteria and glycopeptide-resistant *Enterococcus faecium* outbreaks: a 6-year prospective study in a French multihospital institution, January 2010 to December 2015

**DOI:** 10.2807/1560-7917.ES.2018.23.8.17-00078

**Published:** 2018-02-22

**Authors:** Sandra Fournier, Laure Desenfant, Catherine Monteil, Michèle Nion-Huang, Christian Richard, Vincent Jarlier, the AP-HP Outbreaks Control Group

**Affiliations:** 1 Central Infection Control Team, Assistance Publique-Hôpitaux de Paris, Paris, France; 2 Hôpital Bicêtre, Assistance Publique-Hôpitaux de Paris, Le Kremlin-Bicêtre, France; 3 Sorbonne Universités, UPMC Univ Paris 06, Inserm, Centre d'Immunologie et des Maladies Infectieuses, UMR 1135 & APHP, CHU Pitié-Salpêtrière, Laboratoire de Bactériologie-Hygiène, Paris, France; 4 Members of the AP-HP Outbreaks Control Group are given are the end of the article.

**Keywords:** carbapenemase-producing enterobacteria, glycopeptide-resistant Enterococcus, extensively drug-resistant bacteria, eXDR, outbreaks control, bundle measures

## Abstract

An infection control programme was implemented in a 21,000-bed multihospital institution for controlling the spread of carbapenemase-producing Enterobacteriaceae (CPE) and glycopeptide-resistant *Enterococcus faecium* (GRE), classified as ‘emergent extensively drug-resistant bacteria’ (eXDR) in France. We evaluated factors associated with outbreaks occurrence (n = 103), which followed 901 eXDR introductions (index case followed or not by secondary cases) from 2010 to 2015. In univariate analysis, knowing that patients had been hospitalised abroad, bacterial species (GRE vs CPE, as well as the CPE *Klebsiella pneumoniae* compared with the other Enterobacteriaceae species) and type of measures implemented within the first 2 days of hospitalisation were associated with outbreaks occurrence, but not the type of wards where carriers were hospitalised, nor the eXDR colonisation or infection status. In multivariate analysis, occurrence of outbreaks was significantly lower when contact precautions (odds ratio (OR): 0.34; 95% confidence interval (CI): 0.22–0.54) and even more when dedicated nursing staff (OR: 0.09; 95% CI: 0.02–0.39) were implemented around eXDR index cases within the first 2 days of hospitalisation (p < 10 ^− 3^). GRE introductions were more frequently associated with occurrence of outbreaks than CPE (OR: 3.58; 95% CI: 2.32–5.51, p < 10 ^− 3^). A sustained and coordinated strategy is efficient to limit the spread of eXDR at the scale of a large health institution.

## Introduction

Increase in bacterial resistance is nowadays one of the most important public health issues. Carbapenemase-producing Enterobacteriaceae (CPE) are of particular concerns, since carbapenems represent last line beta-lactam antibiotics for treating patients infected by multidrug-resistant enterobacteria such as those producing extended-spectrum β-lactamases (ESBL) [1,2]. Outbreaks of glycopeptide-resistant *Enterococcus faecium* (GRE) in hospitals are also of concern in many countries [3]. The documented transfer of vancomycin resistance to meticillin-resistant *Staphylococcus aureus* (MRSA) strains raises an additional reason for controlling the spread of GRE [4], especially in countries, such as France, where MRSA rates are still high [5]. Acquired resistance to carbapenems due to carbapenemases in enterobacteria, as well as resistance to vancomycin in *E. faecium*, are, so far, uncommon in France as shown by the European Antimicrobial Resistance Surveillance Network [5]. These multidrug-resistant pathogens share two critical features concerning their dissemination potential: (i) they are hosts of the digestive tract and consequently, are disseminated by faecal route [6], and (ii) their resistant traits are harboured on mobile element, increasing the risk of bacteria to bacteria dissemination. Based on the risk of their dissemination in the general population, these commensal species have been classified as ‘emergent extensively drug-resistant bacteria’ (eXDR) [7] by the French Committee for Public Health, which has published recommendations for preventing their spread in 2013 [8].

Assistance Publique – Hôpitaux de Paris (AP-HP), the largest public healthcare institution in France, has implemented since 2004 a long-term programme for surveillance and control of these eXDR [9-11]. A previous study performed from 2004 to 2012 in this institution showed that the type of measures implemented within the first 2 days, i.e. (i) cohorting eXDR patients and nursing staff dedicated to them, or (ii) contact precautions without cohorting, or (iii) delayed control measures (i.e. use of standard precautions only), significantly influenced the number of secondary cases around a CPE index patient [11]. The objective of the present study was to evaluate at the scale of our whole institution the factors associated with the occurrence of outbreaks around CPE and GRE index cases from January 2010 to December 2015.

## Methods

### Setting

AP-HP is a public health institution administering 38 teaching hospitals (22 acute care and 16 rehabilitation/long-term care (RLTC) hospitals, spread over Paris, suburbs and surrounding counties), with a total of 21,000 beds (10% of all public hospital beds in France) and serving 12 million inhabitants. AP-HP admits 1 million inpatients per year, employs 22,000 physicians, 20,000 nurses and 30,000 assistant nurses. Administrators and medical committees manage AP-HP hospitals locally, but decisions on large investments and general medical policy are taken by the central administration. A local infection control team (LICT) is in charge of prevention and surveillance of healthcare-associated infections in each hospital but decisions of foremost importance for the whole institution are coordinated by a multidisciplinary central infection control team (CICT: infectious disease physician, bacteriologist, epidemiologist and nurse) [9,10,12]. The institutional multidrug-resistant control programme has included, for more than 20 years, different actions such as promotion of contact precautions for MRSA [12] and ESBL-producing Enterobacteriaceae, promotion of alcohol-based hand-rub solutions for hand hygiene, campaigns to decrease antibiotics consumption [10] and implementation of an excreta management policy [13].

### Case definitions

According to national recommendations, eXDR include CPE and GRE [8]. A case was defined as any patient infected or colonised (i.e. positive culture of rectal swab) with an eXDR. An introduction was defined as one index case (the first case identified), followed or not by secondary case(s). A contact patient was defined as any patient whose stay overlapped with the stay of an eXDR case for at least one day in the same unit, or who was cared for by the same personnel as the index case. An outbreak was defined as at least two eXDR cases (i.e. one index case and at least one secondary case among the contact patients) occurring in a given hospital, with a clear epidemiological link (stay during the same period of time in the same unit, or cared for by the same personnel in two different units) and involving indistinguishable eXDR strain based on species, antibiotic susceptibility pattern and mechanism of resistance (carbapenemase or *van* genes).

### Microbiological methods

Microbiological methods to screen patients for GRE and CPE carriage followed French guidelines [14], and were detailed previously [9,11]. Most laboratories used conventional cultures with a turnaround time of 48 hours; some of them added PCR methods. Genotypic methods were used to identify which gene was involved, *vanA* or *vanB* for GRE, *OXA-48-like*, *NDM*, *KPC* or *VIM* for CPE. In case of doubt, strains were sent for further characterisation to the French National Reference Center for Antibiotic Resistance (Kremlin Bicêtre hospital for CPE, Caen hospital for GRE).

### eXDR control programme

The AP-HP eXDR control programme was previously described in detail [9,11]. In short, the most important measures were: 

(i) pre-emptive isolation (contact precautions) and screening for eXDR, of every patient with a history of hospital stay in a foreign country in the past year;

(ii) implementing contact precautions for an eXDR carrier; in addition, it was recommended to dedicate, to the extent possible, nursing staff (one nurse and one caregiver) for the eXDR carrier’s care; if nursing staff could not be dedicated to a single patient, care was organised in the unit by beginning with contact patient’s care and finishing with eXDR patient’s care;

(iii) screening contact patients by culturing rectal swabs and pursuing screening of contact patients once weekly;

(iv) if at least one secondary case was identified, cohorting patients in three distinct areas with dedicated nursing staff: the ‘eXDR patients’ section, the ‘contact patients’ section and the ‘eXDR-free patients’ section for newly admitted patients with no previous contact with eXDR cases.

Control measures were implemented on admission based on the patient history (i.e. recent hospital stay in foreign country) and were respectively continued or cancelled when microbiological results confirmed or infirmed eXDR carriage. In practice, measures in place within the first 2 days following admission of an index case ranged from, at best, quick setting of dedicated nursing staff and contact precautions for the patient, to quick setting of contact precautions without dedicated nursing staff, or to standard (basic level) precautions only if the two types of other control measures were delayed, for example for carriers identified based on a clinical specimen taken during hospitalisation. Thus, each eXDR introduction was classified by CICT and LICTs in the three above categories of measures, the first including the two others and the second including the third.

### Variables explored as possible factors associated with occurrence of outbreaks 

For each introduction, the following factors were collected: histories of previous hospitalisation or stay abroad, type of ward where the index case was admitted (intensive care, surgery, medicine, or RLTC units), categories of measures implemented within the first 2 days of hospitalisation around an index case (dedicated nursing staff, contact precautions or standard precautions), colonisation or infection at the moment of identification of eXDR, species of bacteria (CPE or GRE), and gene resistance involved.

### Statistical analysis

The data were analysed with Stata (version 13, College Station, TX, US). Results are expressed as mean ± standard deviation or median with interquartile range (IQR) for continuous variables or as percentages of the group from which they were derived (categorical variables). A chi-squared test and a Fischer exact test were used to compare categorical variables. Odds ratios (ORs) and 95% confidence intervals (CIs) were calculated for all associations that emerged. Two-tailed tests were used to determine statistical significance; a p value of < 0.05 was considered significant. Multivariate analysis was used to identify independent risk factors for outbreaks’ occurrence. For this analysis, we used logistic regression, and variables found to be significant in univariate testing were incorporated with a stepwise approach. Analyses to identify independent risk factors for occurrence of outbreaks were performed for eXDR introductions and for GRE and CPE introductions separately. A regression analysis was conducted to assess the increase of number of introductions over the 6-year period of the study (Student test). A chi-squared test for linear trend was performed to evaluate the evolution of proportion of outbreaks among introductions (Cochran–Armitage test).

## Results

From January 2010 to December 2015, 901 eXDR introductions, including 655 CPE and 246 GRE, were reported in AP-HP hospitals.

The main species involved in CPE introductions were *Klebsiella pneumoniae* (n = 292, 45%), *Escherichia coli* (n = 273, 42%) and other species (n = 90, 14%). The carbapenemases identified were OXA-48-like (n = 513), NDM (n = 96), KPC (n = 39) and VIM (n = 19). In 12 CPE introductions, two distinct carbapenemases were identified, OXA-48-like and NDM. Among GRE introductions, the genes encoding vancomycin resistance were *vanA* (n = 213) and *vanB* (n = 33).

**Figure 1:**
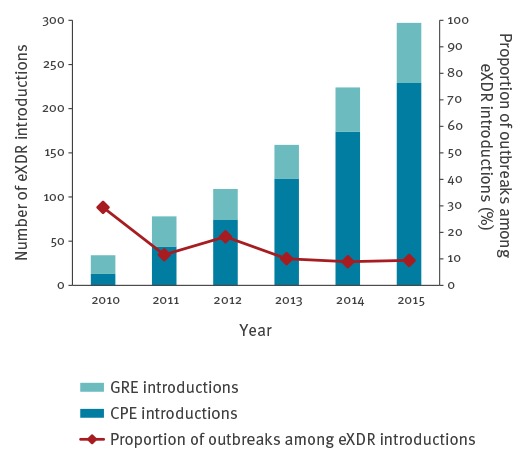
Number of GRE and CPE introductions, and proportion of outbreaks among these eXDR introductions, Assistance Publique–Hôpitaux de Paris, France, 2010–2015 (n = 901 introductions)

CPE: carbapenemase-producing Enterobacteriaceae; eXDR: emergent extensively drug-resistant bacteria; GRE: glycopeptide-resistant *Enterococcus faecium*.

Among the 901 introductions, 669 (74%) involved patients with a known history of hospitalisation abroad (n = 518) or stay abroad (n = 151) within the past year. The number of introductions per year increased significantly over the 6-year period of the study, from 34 in 2010 to 297 in 2015 (p < 0.01). Overall, 103 of the 901 introductions (11%) led to an outbreak. The proportion of outbreaks decreased over time, from 29% (10/34) to 9% (28/397), p < 0.05 (Figure 1).

The median number of outbreaks per hospital was 2, IQR (1–5).

Overall, the 901 eXDR introductions resulted in 1,328 cases. Among these cases, 798 were single cases (i.e. index cases not followed by secondary case) and 530 were clustered in the 103 outbreaks recorded (103 index cases and 427 secondary cases). The number of secondary cases ranged globally from 1 to 46 per outbreak (median 2; IQR: 1–4; mean: 4 ± 6). The median number of secondary cases was 1, IQR (1–4) for CPE outbreaks, and 2, IQR (1–6) for GRE outbreaks respectively.

The characteristics of eXDR introductions and the proportions of outbreaks among these introductions are presented in Table 1. Univariate analysis showed (i) that knowing that a patient had been recently hospitalised or had stayed abroad, and (ii) that dedicated nursing staff and contact precautions around index case within 2 days of hospitalisation were associated with a lower proportion of outbreaks occurrence. Moreover it also showed (iii) that GRE introductions were more frequently associated with an outbreak occurrence than CPE introductions.

**Table 1:** Univariate analysis of variables potentially affecting the proportion of outbreaks among eXDR introductions (CPE or GRE) in the 38 hospitals of Assistance Publique–Hôpitaux de Paris, France, 2010–2015

Variables	Number of eXDR introductions n = 901	Number of outbreaks n = 103	Proportion of outbreaks among introductions	OR (95% CI)	P value
Type of ward					
Surgery	210	17	8%	1	0.19
Intensive care unit	222	25	11%	1.44 (0.75–2.76)	
Medicine	398	49	12%	1.59 (0.89–2.85)	
RLTC	71	12	17%	2.31 (1.04–5.15)	
Known previous hospitalisation or stay abroad in the past year					
Known previous hospitalisation or stay abroad in the past year^a^	669	55	8%	0.34 (0.22–0.53)	< 0.001
Colonisation or infection					
eXDR colonisation	732	81	11%	1	0.34
eXDR infection	169	22	13%	1.27 (0.77–2.09)	
Bacterial species					
CPE	655	51	8%	1	< 0.001
GRE	246	52	21%	3.17 (2.07–4.86)	
Measures implemented around the index case within the first 2 days of hospitalisation					
Standard precautions	367	67	18%	1	< 0.001
Contact precautions	460	34	7%	0.36 (0.23–0.56)	
Dedicated nursing staff	74	2	3%	0.12 (0.03–0.53)	

CI: confidence interval; CPE: carbapenemase-producing Enterobacteriaceae; eXDR: emergent extensively drug-resistant bacteria; GRE: glycopeptide-resistant *Enterococcus faecium*; OR: odds ratio; RLTC: rehabilitation/long-term care.

^a^ The reference is no known hospitalisation abroad in the past year.

Logistic regression analysis showed that measures implemented around the index case were associated with occurrence of outbreaks, as well as bacterial species (Table 2). Indeed, the less stringent the precautions implemented within the first 2 days of hospitalisation, the higher the rate of outbreaks. GRE was also identified as an independent factor associated with outbreaks’ occurrence. Since outbreaks appeared to be more frequent in RLTC wards, the variable RLTC (i.e. hospitalisation in RLTC: yes or no) was also considered in multivariate analysis. Being hospitalised in RLTC was not associated with outbreak occurrence (OR: 1.32; 95% CI: 0.66–2.66, p = 0.44).

**Table 2:** Multivariate analysis of factors associated with occurrence of outbreaks among eXDR introductions (CPE or GRE), in the 38 hospitals of Assistance Publique–Hôpitaux de Paris, France, 2010–2015

Measures implemented around the index case within the first 2 days of hospitalisation	OR (95% CI)	P value
Standard precautions	1	< 0.001
Contact precautions	0.34 (0.22–0.54)	
Dedicated nursing staff	0.09 (0.02–0.39)	
Bacterial species: GRE	3.58 (2.32–5.51)	< 0.001

CI: confidence interval; CPE: carbapenemase-producing Enterobacteriaceae; GRE: glycopeptide-resistant *Enterococcus faecium*; OR: odds ratio.

**Figure 2:**
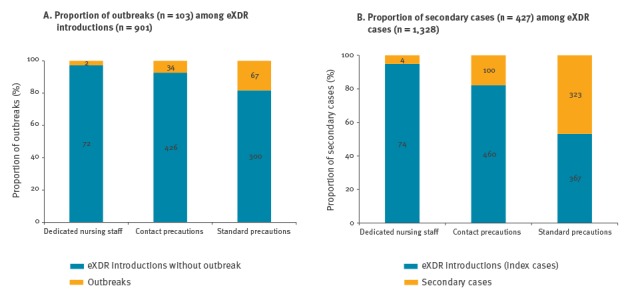
(a) Proportion of outbreaks among eXDR^a^ introductions and (b) proportion of secondary cases among eXDR cases, according to measures implemented within the first two days around an eXDR index case, in the 38 hospitals of Assistance Publique–Hôpitaux de Paris, France, 2010–2015

CPE: carbapenemase-producing Enterobacteriaceae; eXDR: emergent extensively drug-resistant bacteria; GRE: glycopeptide-resistant *Enterococcus faecium*.

^a^ Either CPE or GRE.

Figure 2 illustrates the proportion of outbreaks among eXDR introductions and the number of secondary cases per introduction, according to measures implemented within the first two days.

Univariate (data not shown) and multivariate analysis (Table 3) were also performed separately for CPE and GRE. For both CPE and GRE (Table 3), contact precautions or dedicated nursing staff implemented around the index case were independently associated with the occurrence of outbreaks. Furthermore, for CPE, the species *K. pneumoniae* was independently associated with a higher rate of occurrence of outbreaks.

**Table 3:** Multivariate analysis of factors associated with occurrence of outbreaks among CPE and GRE introductions, Assistance Publique–Hôpitaux de Paris, France, 2010–2015

Measures implemented around the index case within the first 2 days of hospitalisation	OR (95% CI)	P value
CPE		
Standard precautions	1	< 0.001
Contact precautions	0.41 (0.22–0.74)	
Dedicated nursing staff	0.17 (0.02–1.29)	
Bacterial species: *Klebsiella pneumoniae*	4.98 (1.16–21.45)	< 0.05
GRE		
Standard precautions	1	< 0.001
Contact precautions	0.26 (0.13–0.51)	
Dedicated nursing staff	0.05 (0.01–0.40)	

CPE: carbapenemase-producing Enterobacteriaceae; GRE: glycopeptide-resistant *Enterococcus faecium*.

## Discussion

This prospectively designed infection-control intervention analysis reports the largest institutional experience (more than 900 introductions, i.e. index cases, over a period of 6 years) of an eXDR control programme in a country where CPE and GRE are still at an emerging stage (sporadic hospital outbreaks [15]). Bundle measures comparable with those described in the present study (carrier isolation, patient and staff cohorting, active contact tracing and screening) succeeded in containing a large nationwide CPE outbreak in Israel as reported by Schwaber [16,17]. GRE and CPE are commensal bacteria of the digestive tract, prompt to be disseminated by faecal contamination and hand transmission [6]. Identification of efficient measures to control the spread of these organisms in hospitals is of foremost importance to limit healthcare-associated infections for which there are few treatment possibilities [18]. The bundled eXDR programme, implemented since 2004 in our institution, has proven efficient to control GRE outbreaks [9] and CPE spread [11]. The present study confirms that the eXDR programme is efficient to limit the spread of GRE and CPE in our large public health institution during a prolonged period. The number of eXDR index cases dramatically increased in the last years, mostly in relation with previous hospitalisation or stay in foreign countries, and was not the result of changes in number of beds, admissions, rate of occupancy or changes in the organisation of the institution. Despite this increase in index cases, the number of outbreaks decreased and remained contained.

Furthermore, this study allows evaluating factors associated with outbreaks occurrence. Interestingly, no link was found with the type of wards where the carriers were hospitalised. Indeed, outbreaks did not occur more frequently in intensive care units than in medicine or surgery wards, probably because the type of measures implemented is more important than the place where they are implemented. Although the difference was not statistically significant, outbreaks seemed to be a little more frequent in RLTC wards, maybe because eXDR carriers stayed there for a longer period than in acute care wards, and because implementing measures could be more difficult in RLTC wards due to limited human resources.

No difference in the occurrence of outbreaks was found according to the eXDR status, i.e. colonised or infected, of the patient. It is known that patients infected with these digestive commensal bacteria, are generally also colonised and are therefore source of dissemination by faecal route [19].

Interestingly, known hospitalisation or stay abroad was associated with a lower occurrence of outbreaks in univariate analysis. Obviously, these factors are known to favour eXDR carriage but, conversely, knowing that a patient is at risk to be an eXDR carrier allows prompt implementation of control measures at admission, therefore decreasing the risk of cross transmission. In multivariate analysis, the latter factors were not associated with occurrence of outbreaks because they were strongly linked with the type of measures implemented.

The present study allowed to clearly assess the impact on the occurrence of outbreaks of the different types of measures (standard precautions, contact precautions or dedicated nursing staff) implemented during the first 2 days of hospitalisation. Indeed, the rate of outbreaks was lower when nursing staff was dedicated to the index case than when only contact or standard precautions were implemented, as shown by logistic regression analysis. Not only did the occurrence of outbreaks differ according to measures implemented around an index case, but also the size of the outbreaks, the number of secondary cases being higher when only contact or standard precautions were used. Importantly, quickly applying contact precautions around index patients was not sufficient to avoid secondary cases which occurred in 34 of 460 introductions (18/342 CPE, 5% and 16/118 GRE, 14%). This lack of guarantee has been already reported by others [20,21]. Indeed, contact precautions, as well as standard precautions, are not constantly applied with rigor on all days and by all healthcare workers, so they are far from sufficient to avoid transmission of resistant bacteria.

In the present study, dedicated nursing staff and cohorting clearly appeared to be the most effective measure to avoid nosocomial transmission [20,22-25]. However, limitation in nursing staff may be an obstacle to dedicate several healthcare workers to a single index case. If this cannot be done, regular screening of contact patients to rapidly detect secondary cases is critical as well as implementing reinforced outbreak control measures [26]. An evaluation of costs associated with implementation of measures for controlling spread of eXDR in three hospitals of our institution, showed that early identification and implementation of contact precautions was the less expensive scenario [27]. The cost of prevention of outbreaks has to be compared with the cost of controlling an outbreak, particularly when the number of secondary cases is high. Moreover, the cost of preventing spread of eXDR should be balanced with the cost of further eXDR infections in the community.

Interestingly, in multivariate analysis, GRE appeared to spread more easily than CPE. One hypothesis to explain this, is that GRE survives for a prolonged period of time on environmental surfaces and can therefore contaminate more easily healthcare workers’ hands [28]. This hypothesis would encourage to consider a specific protocol for daily and terminal room cleaning after discharge of GRE carriers. It should additionally be emphasised that among CPE introductions, the species *K. pneumoniae* was also independently associated with occurrence of outbreaks.

Our study has potential limitations. First, it was not a randomised, controlled trial aiming at assessing direct causality between measures implemented and outcome. The rapid spread of eXDR in neighbouring countries [5] triggered quick and strong actions to control this emerging problem in France, contraindicating randomised comparative studies. Most studies reporting GRE or CPE control programmes assessed the effect of introducing a bundle of interventions, which made it difficult to determine the effectiveness of individual measures [16,20,22,26,29,30]. Our study allows comparing the impact of different levels of control measures (standard precautions, contact precautions, dedicated nursing staff) on the occurrence of outbreaks and the size of outbreaks, using a pragmatic approach in real life.

Another limitation of the study is that outbreaks were defined based on epidemiological and microbiological criteria and not on the genotype of the strains involved. Since eXDR introductions were rare in our institution and most index cases had a history of stay abroad, we considered pragmatically that cases, that occurred in the same unit than the index case, as well as in the same period of time and with the same species, same gene of resistance and same antibiotic susceptibility pattern, were linked to the index case, without other molecular considerations.

A further limitation of our study is that we did not evaluate hand hygiene compliance, the most important measure to prevent and control hospital-associated infection, at the level of the unit where eXDR cases were admitted. Hand hygiene with alcohol-based hand-rub solutions was reinforced around eXDR carriers, but we were not able to integrate alcohol hand rub solution consumption in the statistical analysis, since the consumption is not measured at the ward level but only at hospital level in our institution. Management of excreta (stools and urines) is another point of major importance to control spread of faecal bacteria in hospitals. Healthcare workers were asked to be especially vigilant on hand hygiene during excreta management and were encouraged to use disposable excreta collection bags for every eXDR carrier requiring the use of a bedpan. Unfortunately this point was not taken in account in our statistical analysis.

In conclusion, this study shows that CPE and GRE spread can be strongly limited by a specific control programme, even at the scale of a large multihospital institution. The level of measures implemented around index case within the 2 days following hospitalisation was associated with the occurrence of outbreaks, dedicated staff being more efficient than contact precautions which were in turn more efficient than standard precautions. Such a programme requires strong and sustained involvement of all stakeholders, particularly the infection control team, medical and nursing staff, microbiologists and hospital administrators. Our results could help to convince and stimulate all these stakeholders.

## Notes

**Members of The AP-HP Outbreaks Control Group:** Florence Espinasse, Marie-Christine Gramer, Jean-Louis Gaillard, Beate Heym, Hôpital Ambroise Paré, AP-HP, Boulogne, France; Véronique Derouin, Florence Doucet-Populaire, Hôpital Antoine Béclère, AP-HP, Clamart, France; Nathalie Bourlon, Etienne Carbonnelle, Anne Jacolot, Delphine Seytre, Hôpital Avicenne, AP-HP, Bobigny, France; Simone Nerome, Marie-Hélène Nicolas-Chanoine, Isabelle Garrigues, Hôpital Beaujon, AP-HP, Clichy, France; Nicolas Fortineau, Audrey Leon, Patrice Nordmann, Souad Ouzani, Daniel Vittecoq, Hôpital Bicêtre, AP-HP, Kremlin-Bicêtre, France; Antoine Andremont, Laurence Armand-Lefevre, Gisèle Bendjelloul, Gabriel Birgand, Christine Bonnal, Isabelle Lolom, Jean-Christophe Lucet, Hôpital Bichat, AP-HP, Paris, France; Francois-Xavier Chedhomme, Françoise Lion, Hôpital Broca, AP-HP, France; Laurence Drieux-Rouzet, Hôpital Charles Foix, AP-HP, Ivry Sur Seine, France; Véronique Moulin, Hôpital Corentin Celton, AP-HP, Paris, France; Marie-Thérèse Baixench, Hervé Blanchard, Anne Casetta, Leonie Meyer, Hélène Poupet, Claire Poyart, Hôpital Cochin, AP-HP, Paris, France; Anani Akpabie, Hôpital Emile Roux, Limeil-Brévannes, France; Najiby Kassis-Chikhani, Anne Carbonne, Bruno Hacquin, Jean-Luc Mainardi, Guy Meyer, Frederique Mignot, Isabelle Podglajen, Hôpital Européen Georges Pompidou, AP-HP, Paris, France; Akram Kabani, Hôpital Georges Clémenceau, AP-HP, Champcueil, France; Nicole Haber, Hôpital Adélaïde Hautval, AP-HP, Paris, France; Christian Brun-Buisson, Jean-Winoc Decousser, Chloé Jansen, Sarah Jolivet, Philippe Lesprit, Hôpital Henri Mondor, AP-HP, Créteil, France; Isabelle Durand, Hôpital Jean Verdier, AP-HP, Bondy, France; Nadine Sabourin, Hôpital Joffre-Dupuytren, AP-HP, Draveil, France; Rishma Amarsy, Emmanuelle Cambau, Valérie Chauvin, Karine Jan, Laurent Raskine, Pierre-Olivier Sellier, Hôpital Lariboisière, AP-HP, Paris, France; Catherine Branger, Géraldine Marcadé, Vincent Fihman, Beatrice Hedoux-Queau, Jean-Philippe Tabut, Jean-Damien Ricard, Hôpital Louis Mourier, AP-HP, Colombes, France; Pierre Frange, Sandrine Gotty, Patricia Husson, Isabelle Le Guinche, Xavier Nassif, Jean-Ralph Zahar, Hôpital Necker, AP-HP, Paris, France; Patricia Baune, Vanessa Prat, Christian Sengelin Hôpital Paul Brousse, AP-HP, Villejuif, France; Claudio Garcia Sanchez, Martine Jarry, Jocelyne Leconte, Nathalie Osinski, Jérôme Robert, Corinne Tamames, Hôpital Pitié-Salpêtrière, AP-HP, Paris, France; Jean-Louis Herrmann, Christine Lawrence, Margaux Lepainteur, Arzu Tackin, Hôpital Raymond Poincaré, AP-HP, Garches, France; Stéphane Bonacorsi, Catherine Doit, Kamila Belhacel, Valérie Souyri, Hôpital Robert Debré, AP-HP, Paris, France; Frédéric Barbut, Dominique Decré, Marie Jeanne Kosmann, Françoise Truchot, Hôpital Saint Antoine, AP-HP, Paris, France; Isabelle Simon, Hôpital Sainte Périne, AP-HP, Paris, France; Martine Rouveau, Micheline Thégat, Hôpital Saint Louis, AP-HP, Paris, France; Guillaume Arlet, Daniel Bonnet, Michel Denis, Hôpital Tenon, AP-HP, Paris, France; Béatrice Salauze, Béatrice Quinet, Hoang Vu Thien, Géraldine Widiez, Hôpital Trousseau, AP-HP, Paris, France; Monique Duviquet, Emeline Granier-Nauge, Sonia Teixeira, Hôpital Vaugirard, AP-HP, Paris, France; Alexandra Lomont, Cyril Meloni from the Central Infection Control Team, AP-HP, Paris, France.
